# Inhibition of Carrageenan-Induced Cutaneous Inflammation by PPAR Agonists Is Dependent on Hepatocyte-Specific Retinoid X Receptor*Alpha*

**DOI:** 10.1155/PPAR/2006/96341

**Published:** 2006-07-05

**Authors:** Yu-Jui Yvonne Wan, Mostafa Z. Badr

**Affiliations:** ^1^Department of Pharmacology, Toxicology & Therapeutics, The University of Kansas Medical Center, Kansas City, KS 66160, USA; ^2^Division of Pharmacology, School of Pharmacy, University of Missouri-Kansas City, Kansas City, MO 64108, USA

## Abstract

It has been proposed that PPAR-dependent, accelerated catabolism of proinflammatory mediators may contribute to the fast resolution of inflammation. Because retinoid X receptors are obligate heterodimer partners of PPARs, we investigated the impact of deleting hepatocyte-specific RXRα on the antiedema effect of PPAR agonists. In wild-type mice (WT), pretreatment with the PPARα agonist perfluorooctanoic acid diminished carrageenan-induced paw edema by 66 ± 10%. This effect was essentially absent (13 ± 8%) in hepatocyte-specific RXRα-deficient mice. Similarly, pretreatment of WT mice with the PPARδ agonist L-783483 or the PPARγ agonist L-805645 caused 54 ± 1% and 38 ± 8% reduction in carrageenan-induced paw edema, respectively. These effects were also significantly diminished or absent in hepatocyte-specific RXRα-deficient mice. In contrast, aspirin reduced carrageenan-induced paw edema equally in WT and hepatocyte-specific RXRα-deficient mice. The identification of RXRα as an important factor involved in the antiedema effect produced by agonists of the three PPAR subtypes is a significant achievement towards the goal of designing novel, effective anti-inflammatory drugs.

## INTRODUCTION

It has been reported by us [1–3], as well as by others [4–6] that agonists of the peroxisome proliferator-activated receptors (PPARs) diminish inflammatory responses. In attempting to delineate mechanisms involved in this effect, it was proposed among various other postulates that termination of the biological activity of proinflammatory mediators might occur in the liver *via* PPAR-dependent catabolic pathways [4]. These pathways are known to be augmented following exposure to PPAR agonists, particularly those which activate PPARα [4].

Retinoid X receptor *alpha* (RXRα) is reported to play a central role in mediating PPAR-induced effects [7–9], and the expression of both PPARs and RXRs decreases in tissues, including the liver of animal models of inflammation [10]. Therefore, we hypothesized that the absence of hepatocyte-specific RXRα shall interfere with the ability of PPAR agonists to control inflammation. Using the intraplantar injection of carrageenan as a well-established in vivo model of inflammation, the present experiments were designed to evaluate the anti-inflammatory effect of subtype-selective PPAR ligands in hepatocyte-specific RXRα-deficient mice. Results show that hepatocytic RXRα plays an important role in mediating the PPAR-induced amelioration of cutaneous inflammation. We postulate that the hepatocyte-specific RXRα is involved in enhancing the metabolic removal of proinflammatory mediators and/or increasing the production of anti-inflammatory mediators in the liver.

The identification of a specific receptor subtype (RXRα) in a specific cell population (hepatocytes) may prove instrumental in the pursuit to fully elucidate mechanisms involved in the antiinflammatory effect of PPAR agonists, as well as invaluable to the effort of developing novel effective anti-inflammatory drugs.

## MATERIALS AND METHODS

### Animals and materials

Male hepatocyte-specific RXRα-deficient mice were produced as reported previously [9]. Animals were cared for in accordance to the guidelines set forth by the National Institutes of Health regarding the proper treatment and use of laboratory animals. All experiments were approved by the University of Missouri–Kansas City Institutional Animal Care and Use Committee.

Carrageenan (type IV, *lambda*) was obtained from Fluka (Milwaukee, WI), while aspirin and perfluorooctanoic acid were from Sigma Aldrich (St Louis, MO). The PPARγ agonist L-805645 and PPARδ agonist L-738483 were provided by Merck (Rahway, NJ).

### Animal treatment

Tested compounds (200 μL in normal saline at a dose of 100 mg/kg), or the saline vehicle, was administered intraperitoneally to wild-type and hepatocyte-specific RXRα-deficient mice. Since we previously observed that PPAR agonists equally diminished inflammation when given 30 minutes prior to, or 90 minutes after the induction of paw edema [1], we limited the present study to the 30 minute *pre*-carrageenan injection of PPAR agonists. Carrageenan (30 μL of 1% in normal saline), or an equivalent volume of saline, was subcutaneously injected into the midplantar region of the right hindpaw. Carrageenan-induced edema, reflected as an increase in dorsal-to-ventral paw thickness, was measured with a microcaliper (Mitutoyo Corp; Kanagawa, Japan). The average of 3 measurements at each time-point was calculated.

### Data analysis and statistics

**Figure 1:**
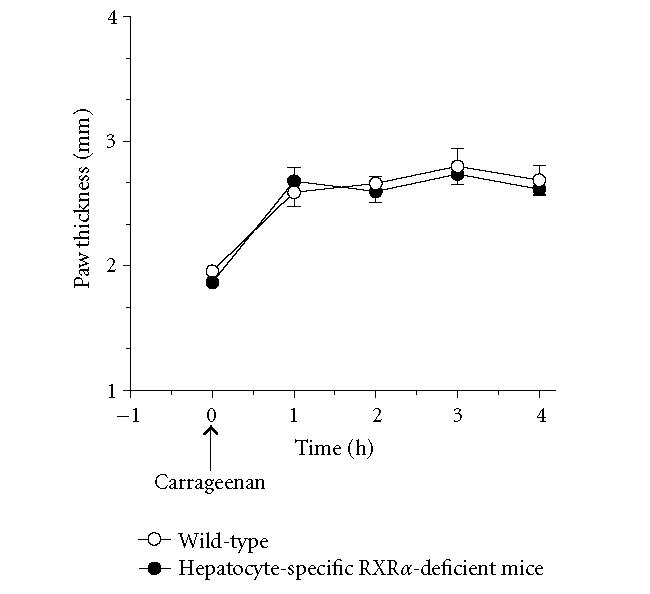
*Carrageenan-induced paw edema in hepatocyte-specific RXRα-deficient mice.* Wild-type and hepatocyte-specific RXRα-deficient mice were treated with 30 μL of 1% carrageenan in normal saline subcutaneously into the midplantar region of the right hindpaw. Carrageenan-induced edema, reflected as an increase in dorsal-to-ventral paw thickness, was measured with a microcaliper. Values represent mean ±SEM. *n* = 8 mice per group.

**Figure 2:**
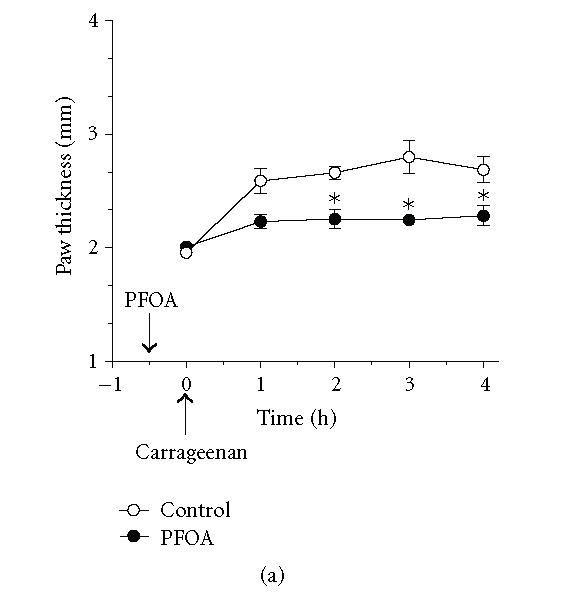

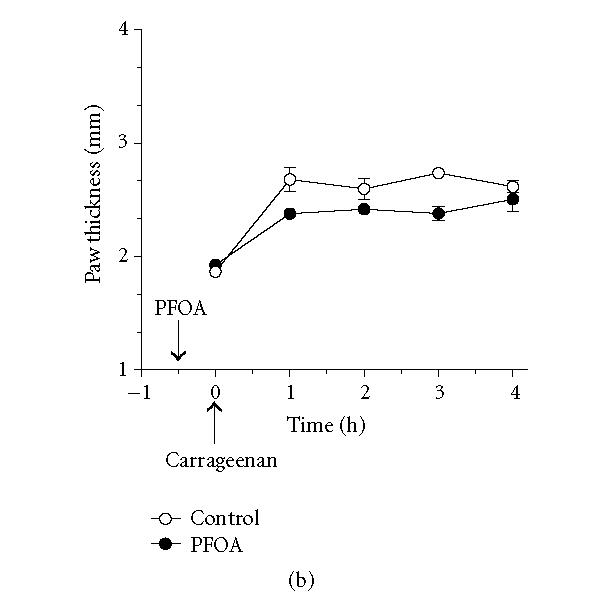
*Effect of the PPARα agonist perfluorooctanoic acid (PFOA) on the temporal profile of carrageenan-induced paw edema in wild-type and hepatocyte-specific RXRα-deficient mice.* Wild-type (A) and hepatocyte-specific RXRα-deficient mice (B) were treated intraperitoneally with (100 mg/kg) of PFOA, 30 minutes before induction of inflammation. As indicated by the arrow, carrageenan (1%, intraplantar) was injected at *t* = 0. Carrageenan-induced edema is reflected by an increase in paw thickness. Values represent mean ±SEM. *n* = 4 mice per group. *P < .05 versus corresponding wild-type point.

**Figure 3:**
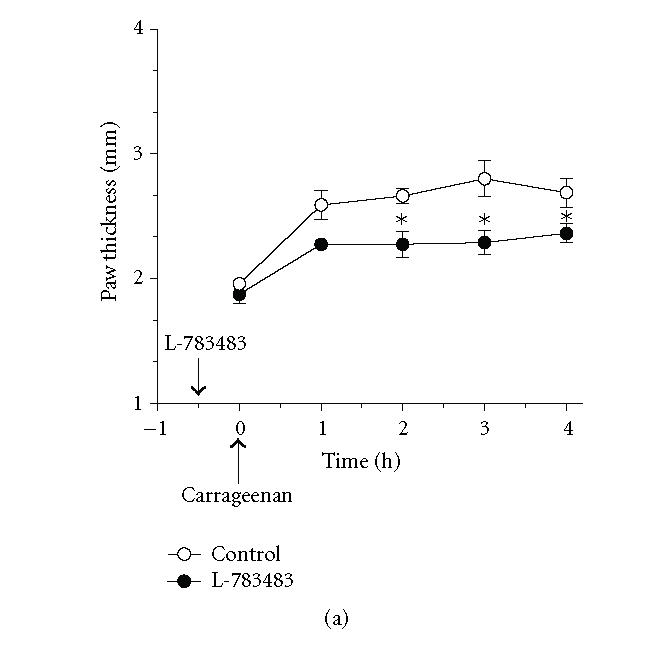

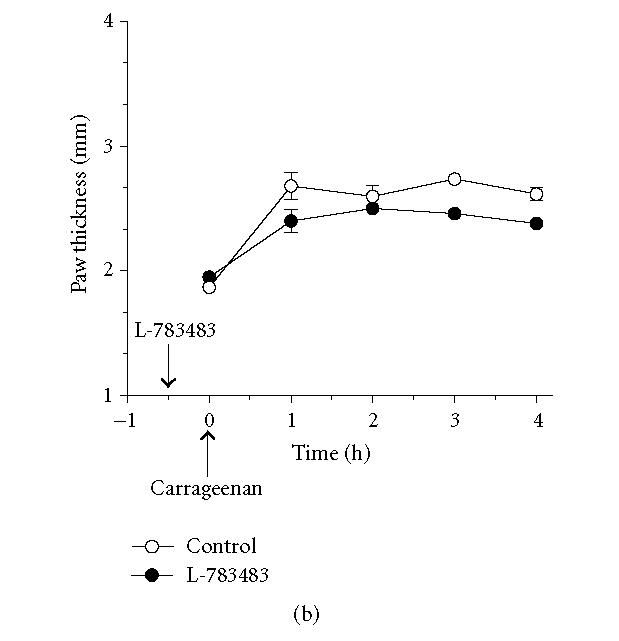
*Antiedema effect of the PPARδ agonist L-738483 on carrageenan-induced paw edema in wild-type and hepatocyte-specific RXRα-deficient mice.* Wild-type (a) and hepatocyte-specific RXRα-deficient mice (b) were treated intraperitoneally with (100 mg/kg) of L-738484, 30 minutes before induction of inflammation. As indicated by the arrow, carrageenan (1%, intraplantar) was injected at *t* = 0. Carrageenan-induced edema is reflected by an increase in paw thickness. Values represent mean ±SEM. *n* = 3–5 mice per group. *P < .05 versus corresponding wild-type point.

**Figure 4:**
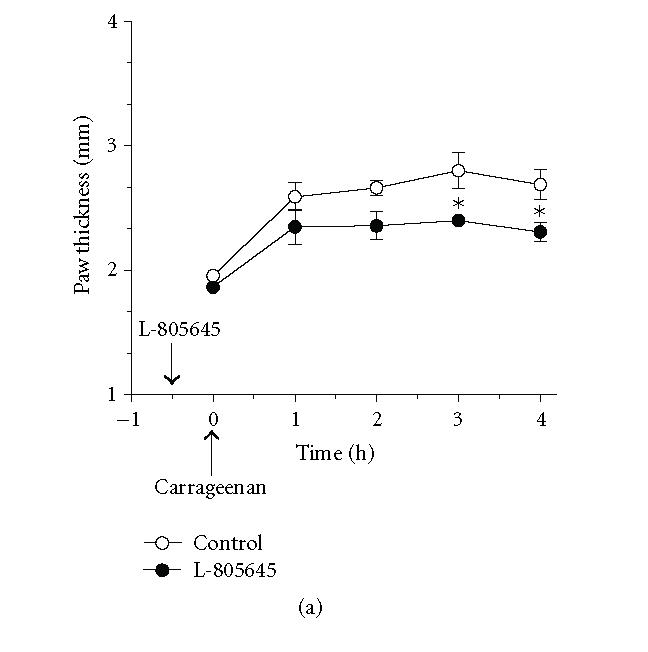

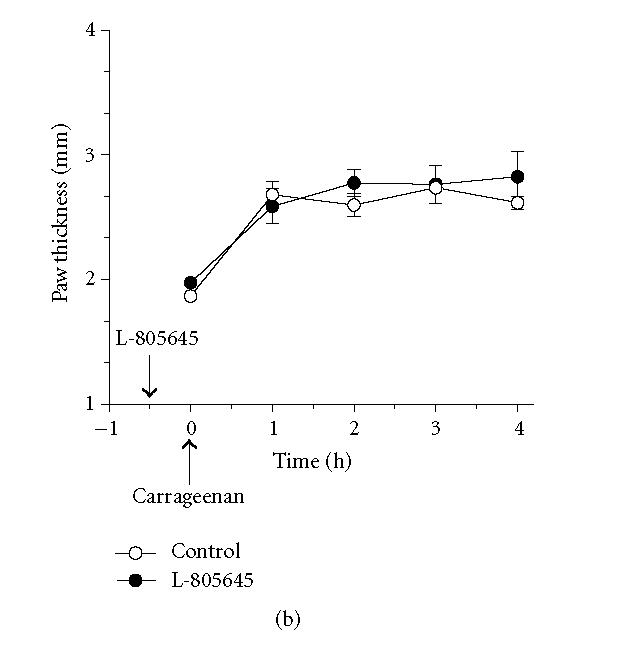
*Effect of the PPARγ agonist L-805645 on the profile of carrageenan-induced paw edema in wild-type and hepatocyte-specific RXRα-deficient mice.* Wild-type (a) and hepatocyte-specific RXRα-deficient mice (b) were treated intraperitoneally with (100 mg/kg) of L-805645, 30 minutes before induction of inflammation. As indicated by the arrow, carrageenan (1%, intraplantar) was injected at *t* = 0. Carrageenan-induced edema is reflected by an increase in paw thickness. Values represent mean ±SEM. *n* = 4–6 mice per group. *P < .05 versus corresponding wild-type point.

**Figure 5:**
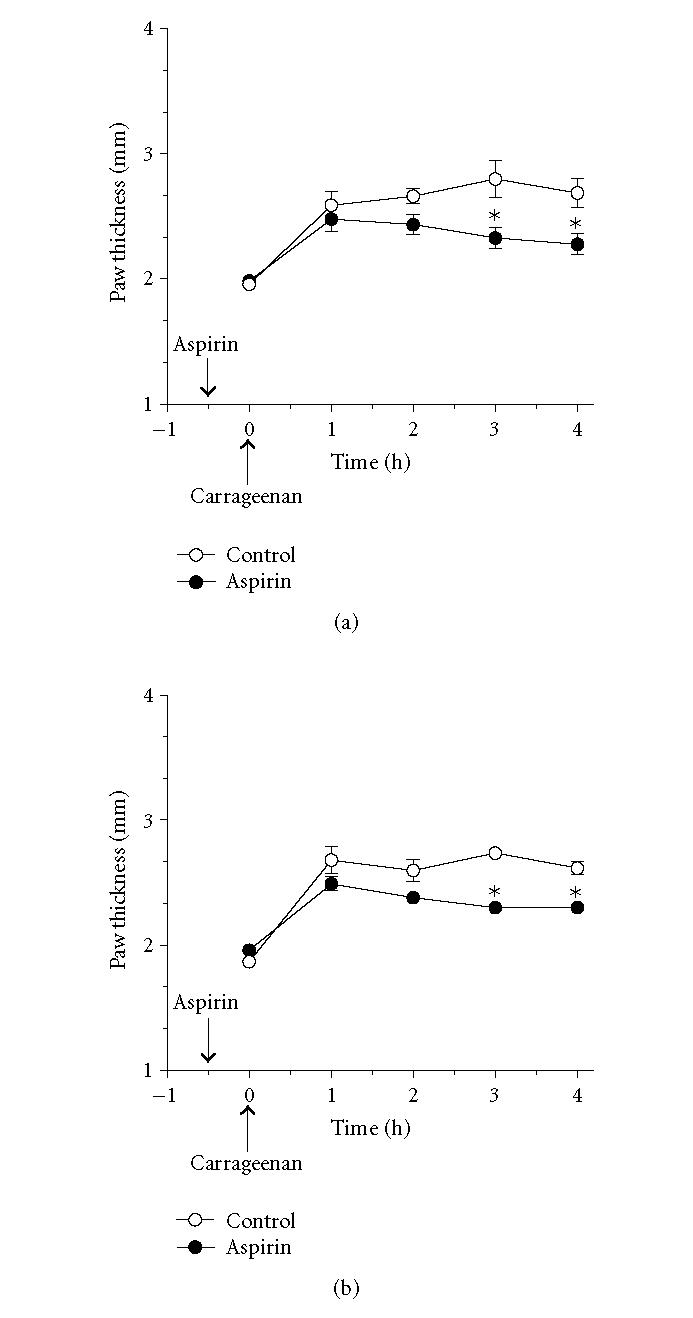
*Effect of aspirin on carrageenan-induced paw edema in wild-type and hepatocyte-specific RXRα-deficient mice.* Wild-type (a) and hepatocyte-specific RXRα-deficient mice (b) were treated intraperitoneally with (50 mg/kg) of aspirin, 30 minutes before induction of inflammation. As indicated by the arrow, carrageenan (1%, intraplantar) was injected at *t* = 0. Carrageenan-induced edema is reflected by an increase in paw thickness. Values represent mean ±SEM. *n* = 3–5 mice per group. *P < .05 versus corresponding wild-type point.

**Figure 6:**
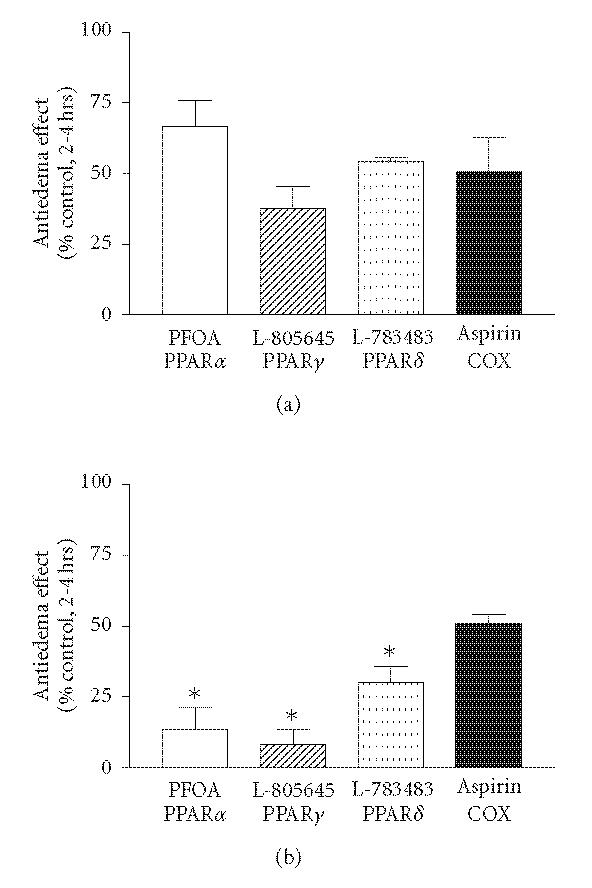
*Percent inhibition of paw edema by various PPAR agonists and aspirin in wild-type (a) and hepatocyte-specific RXRα-deficient (b) mice.* Data shown in Figures 2–5 above are expressed as the percentage change in paw edema relative to control group 2–4 h after carrageean injection. Values represent mean ±SEM. *N* = 3–6 mice per group. *P < .05 versus corresponding wild-type group.

Carrageenan-evoked edema responses were analyzed by Student's *t* test (Figures 1 and 6), or ANOVA (Figures 2–5). *P* < .05 was considered significant.

## RESULTS

### Influence of hepatocyte-specific RXRα on carrageenan-induced paw edema

As illustrated in Figure 1, intraplantar injection of carrageenan in wild-type mice increased paw thickness, with a peak at approximately 1 hr post-injection. The maximal increase in paw thickness was 35% over basal values, and lasted through the 4 hr duration of the experiment. Mice lacking hepatocyte-specific RXRα also showed a comparable increase and trend in paw thickness in response to intraplantar carrageenan injection (Figure 1).

### Antiedema effect of PPAR agonists in hepatocyte-specific RXRα-deficient mice

In wild-type mice, pretreatment with 100 mg/kg of the PPARα agonist perfluorooctanoic acid (PFOA) diminished carrageenan-induced paw edema by 66 ± 10% 2–4 h following carrageenan injection (Figures 2(a) and 6). This effect was essentially absent (13 ± 8%) in hepatocyte-specific RXRα-deficient mice (Figures 2(b) and 6). Similarly, pretreatment of wild-type mice with similar doses of either of the PPARδ agonist L-783483, or the PPARγ agonist L-805645 caused 54 ± 1% and 38 ± 8% reduction in carrageenan-induced paw edema, respectively (Figures 3(a), 4(a), and 6). These effects were significantly lower (30 ± 6%) or absent (8 ± 5%) in hepatocyte-specific RXRα deficient mice, respectively (Figures 3(b), 4(b), and 6). Conversely, aspirin which is a known inhibitor of the cyclooxygenase enzyme, reduced carrageenan-induced paw edema equally in wild-type and hepatocyte-specific RXRα-deficient mice (Figures 5 and 6). The reduction in paw edema was 51 ± 12% and 51 ± 3% x in these two groups of mice, respectively (Figure 6).

## DISCUSSION

Retinoid X receptors (RXR) play a central role as obligate heterodimer partners for numerous nuclear receptors, including PPARs [7]; thereby serving as master regulators of crucial pathways [7]. The RXR/PPAR heterodimer is activated by agonists of either RXR or PPAR, leading to the activation of specific pathways [8], with the combination of RXR and PPAR agonists causing a greater effect than agonists of either receptor alone [8]. Genetic deletion of hepatocyte-specific RXRα drastically, albeit not completely, interfered with PPAR-mediated pathways [9, 10]. In light of these facts, this study was pursued to take advantage of the availability of mice lacking hepatocyte RXRα to investigate potential role played by the liver in the antiedema effect ascribed to PPAR agonists.

Our data clearly show that the absence of hepatocyte-specific RXRα significantly diminished the ability of agonists of PPARα, PPARδ, and PPARγ to exert antiedema effect in vivo, while exhibiting no effect on aspirin-induced antiedema (Figures 2–6). A previous study concluded that activation of PPARα hastened the catabolic termination of the inflammatory response to leukotriene B_4_ [4]. In support of this conclusion, preliminary experiments by the same authors showed diminished plasma clearance of leukotriene B_4_ in PPARα-deficient mice, as compared to wild-type mice [4]. It should be noted however that in contrast to the beneficial effect of PPARα agonists in controlling inflammatory processes, published reports present evidence for a proinflammatory response to these agonists; an effect that is explained by potential differences in cell- and/or tissue-specific susceptibility [11].

PPARγ agonists have been shown to interfere with numerous pathways involved in the inflammatory process, in the course of exerting their reported anti-inflammatory activity [11]. However, whether agonists of PPARγ and/or PPARδ recruit the hepatic metabolic machinery to control inflammation is not reported in the literature. Thus, this is the first report, to our knowledge, that presents an evidence for a unifying mechanism involving the liver, explaining the anti-inflammatory effect of agonists of the three known PPAR subtypes.

The specific requirement for RXRα by *all* PPAR subtypes, observed in this study, may be explained by the fact that this receptor is a heterodimer partner of the three PPAR subtypes. The absence of RXRα is consequently expected to hinder the activation of pathways controlled by agonists selective to any of the various PPAR subtypes. In order to explain the residual anti-inflammatory effect of PPAR agonists observed in hepatocyte-specific RXRα-deficient mice, we propose the following. (1) It is noteworthy that these agonists have been shown to produce effects that are not PPAR-dependent [12–16]. This is an unlikely explanation however in light of the fact that preliminary experiments in our laboratories reveal that PPARα plays a significant role in mediating the anti-inflammatory effect of its agonists (not shown). (2) Another possibility that may explain the residual anti-inflammatory effect of PPAR agonists observed in hepatocyte-specific RXRα-deficient mice takes into considerations the fact that these mice retain the ability to express RXRβ and RXRγ in hepatocytes [9, 10]. Both of these receptor subtypes may act as heterodimer partners of PPARs in the absence of RXRα [9, 10]. (3) Since our animal model lacks RXRα exclusively in hepatocytes [10], RXRα present in liver cell types other than hepatocytes may contribute to the partial anti-inflammatory effect of PPAR agonists observed in these mice. (4) Lastly, whether there are PPAR-mediated effects that are not fully dependent on the heterodimerization of these receptors with RXRs is an interesting postulate that deserves evaluation.

In conclusion, data presented here show that hepatic events involving RXRα are crucial to the antiedema effect of PPAR agonists. These effects may include acceleration of the metabolic removal of proinflammatory mediators and/or enhanced production of antiinflammatory mediators in the liver. Identifying hepatocyte-specific RXRα as a crucial link in the mechanism by which PPAR agonists produce anti-inflammatory effect is indeed a significant first step towards fully defining molecular events involved in this action, as well as designing novel, effective anti-inflammatory drugs.
